# Malleostapedotomy with the self-fixing and articulated titanium piston

**DOI:** 10.1007/s00405-018-4999-z

**Published:** 2018-05-19

**Authors:** J. Burggraaf, E. A. M. Mylanus, R. J. E. Pennings, Cor Cremers

**Affiliations:** Department of Otorhinolaryngology, Donders Institute for Brain, Cognition and Behaviour, Radboud University Medical Center, PO Box 9101, 6500 HB Nijmegen, The Netherlands

**Keywords:** Otosclerosis, Tympanosclerosis, Stapes surgery, Hearing loss, Malleostapedotomy, Kurz–Häusler articulated titanium piston

## Abstract

**Objective:**

To analyze the results of malleostapedotomy performed by applying the self-fixing and articulated titanium piston according to Häusler.

**Study design:**

Retrospective case review.

**Setting:**

Tertiary referral center.

**Patients and interventions:**

This study concerns a retrospective analysis of the results of malleostapedotomy with the use of a self-fixing articulated titanium piston in 16 ears of 16 consecutively treated patients between 2005 and 2009. The medical files were used for the acquisition of data on medical and surgical history and to obtain pre- and postoperative audiometry. Diagnosis and outcomes of mainly revision surgeries are presented and compared to the literature.

**Main outcome measures:**

Effect of (revision) malleostapedotomy by evaluating postoperative audiometry and air–bone gap closure.

**Results:**

The postoperative air–bone gap closure was ≤ 10 dB in 9/16 (56%) ears and within ≤ 20 dB in 13/16 (81%) ears. The mean postoperative air–bone gap was 14.3 dB HL (0.5–2.0 kHz) and 17.3 dB HL (0.5–4.0 kHz). Postoperatively, there was no increase in bone conduction thresholds larger than 3 dB (0.5–2.0 kHz) and postoperative dizziness was absent or very limited and transient.

**Conclusions:**

The malleostapedotomy procedure has become surgically less demanding over time by the technical improvements present in the nowadays available pistons. The design of the self-fixing and articulated titanium piston used in the present group of patients allows a safe and straight-forward malleostapedotomy procedure. Present hearing outcomes match with results presented in the literature.

**Electronic supplementary material:**

The online version of this article (10.1007/s00405-018-4999-z) contains supplementary material, which is available to authorized users.

## Introduction

The malleovestibulopexy procedure was originally a complicated surgical procedure which even in the most experienced hands has been reported to have a relatively high percentage of serious complications and deteriorations (1–10%) at the level of the inner ear [1–4]. Over time, less invasive and, therefore, potentially less risky surgical procedures have been developed in case of a fixed stapes footplate and an absent or eroded incus that precludes fixation of a piston.

**Fig. 1:**
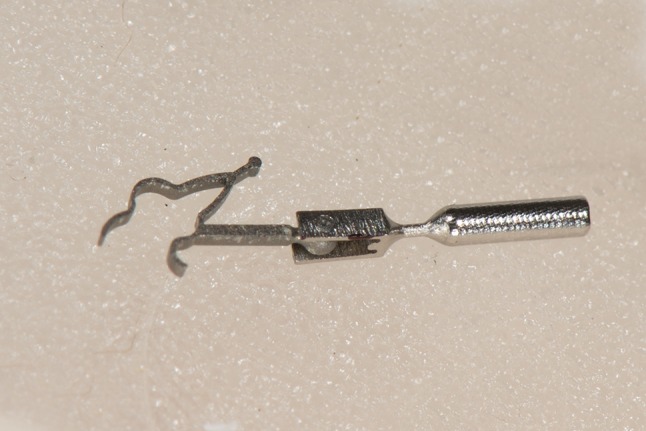
The self-retaining and self-fixing titanium, malleostapedotomy clip piston according to Häusler [7]

Following the gradual introduction of the incudostapedotomy procedure replacing the incudostapedectomy in the eighties of the previous century, the smaller opening of the footplate also became the trend in malleostapedotomy procedures [5, 6]. The full titanium self-fixing malleus grip and articulated piston, introduced in 2004 by Häusler, renders access to the stapedotomy opening. Both the clip and the mobility at the level of the joint facilitate a more soft and gentle handling at the level of the vestibule than the conventional straight design piston [6, 7]. An image of the piston is provided in Fig. 1.

The malleostapedotomy procedure has always been regarded as a most delicate and even last resort option to be applied, preferably by the most experienced hands. Surgical series in the literature, almost all quite limited in numbers, originated from mostly well-known otological centers (Table 1). The surgical outcomes for hearing and balance in the present series with the self-fixing articulated piston are presented below and compared to data from the literature on malleostapedotomy.

**Table 1:** Comparison of results after malleostapedotomy

Author/year (reference)	City of origin	No. of ears	AB-gaps ≤ 10 dB	AB-gaps ≤ 20 dB	Postoperative hearing improvement (%)	Type of piston	Frequencies (kHz)
Sheehy 1982 [1]	Los Angeles	146	51%	81%	84	Stainless steel wire loop prosthesis	–
Shea 1983 [2]	Memphis	35	–	(84%)	–	Malleus all-Teflon prosthesis	–
Schuknecht 1986 [3]	Boston	187	36%	69.1%	–	Schuknecht prosthesis	–
Eberle (Fisch) 1992 [11]	Zürich	123	–	–	90	–	–
Tange 1996 [12]	Amsterdam	41	(70%)	(88%)	93	Schuknecht prosthesis	–
Oestreicher and Häusler 1998/2000/2004 [4, 13, 14]	Bern	39/40	(40%)	(85%)	92	Schuknecht prosthesis	0.5–3.0
Fisch et al. 2001 [15]	Zürich	56	(18%)	(77%)	–	Fisch-Storz titanium prosthesis	–
Kohan and Sorin 2003 [16]	New York	5	(60%)	(100%)	–	Standard wire-Teflon prosthesis	0.5–3.0
Sarac et al. 2006 [17]	Boston	33	(44%)	(72%)	–	Schuknecht prosthesis	–
Dalchow et al. 2007 [18]	Marburg	6	(66%)	(83%)	–	Fisch-Storz titanium prosthesis	–
Häusler and Steinhart 2007 [7]	Bern	10	–	(100%)	–	Kurz-Häusler articulated clip prosthesis	–
Kisilevski et al. 2009 [19]	Toronto	7 (cong. ears)	4/7 (= 57%)	5/7 (= 71%)	–	Causse large loop Piston and Cawthorne prosthesis	–
Kisilevski et al. 2009 [20]	Toronto	24	(33%)	(61%)	–	Causse large loop Piston and Cawthorne prosthesis	–
Gluth et al. 2011 [21]	Perth	7	(43%)	(71%)	–	Stryker–Leibinger titanium stapes piston	0.5–3.0
Rambousek et al. 2012 [22]	Luzern	60 (28 + 32)	Primary *N* = 28 (61%) Revision *N* = 32 (38%)	Primary *N* = 28 (100%) Revision *N* = 32 (81%)	–	Fisch-Storz titanium prosthesis	0.5–2.0 0.5–3.0 0.5–4.0
Chang et al. 2012 [25]	Seoul	14 (7 + 7)	Handle (29%) Neck malleus (43%)	Handle (57%) Neck malleus (86%)	–	Platinum wire piston	0.5–3.0
Magliulo et al. 2013 [23]	Rome	14	5 (= 36%)	11 (= 79%)	–	Nitinol prosthesis	–
This series 2015	Nijmegen	16	(56%)	(81%)	–	Kurz–Häusler articulated clip prosthesis	0.5–2.0 0.5–4.0

## Patients and methods

In this retrospective study, the results obtained in 16 consecutive malleostapedotomies using the CliP® Piston MVP Häusler Design from Kurz were evaluated. The surgical procedures were performed in the period from January 2005 to July 2009. Five cases were excluded from evaluations: three cases with congenital minor ear anomalies and two cases with severe mixed hearing loss. In one of those two cases with a most severe mixed hearing loss, hearing after revision surgery gradually deteriorated to total deafness. One of the other excluded cases (case VI: 8), has been presented as part of a report on a new syndrome (Ref. [8]).

Included were eleven patients with otosclerosis, one patient with osteogenesis imperfecta and four patients with a history of chronic otitis media and tympanosclerosis. In all 16 patients, a fixed footplate was observed at previous or at revision surgery. It concerned 10 males and 6 females. Ages varied between 25 and 58 years (median age of 51 years).

In all but one of the surgical procedures, a transcanal approach with a flexible speculum holder and an aural speculum was used. In only one surgery, an endaural widening incision with canalplasty of the external auditory canal (bony widening of the ear canal) was applied. In this case, limited exposure precluded the use of the speculum holder. No cement or other material was used, either to improve fixation at the level of the malleus or to prevent extrusion of the piston on the level of the footplate. The surgical transcanal method using an aural speculum holder was well described by Häusler and Steinhart [7]. They describe the application of some fat from the ear lobe around the piston at the level of the footplate. Besides this, some bone cement was applied on the malleus to secure additional fixation of the malleus clip piston to the malleus. These extra fixations have also been reported by Häusler in figures in his review contribution on progresses made in stapes surgery [9]. Fisch [10] showed already in 1994 by figures that he secured in his malleostapedotomy procedure the fixation of the piston to the vestibule by placing connective tissue on the footplate around the piston. In addition, blood and fibrin glue were applied around to secure that fixation [10]. He named this procedure “sealing of stapedotomy opening” and has also been applied in other types of stapes surgery. In our surgical series, these extra opportunities to help to secure the fixation of the malleostapedotomy piston have not been applied.

The pre- and postoperative hearing thresholds for bone and air conduction were acquired at 0.5, 1.0 and 2.0 kHz and for 0.5, 1.0, 2.0 and 4.0 kHz, respectively. Routinely there were no data available for 3.0 kHz. The lack or presence of some direct postoperative dizziness had been noted for every case in their medical files and will be provided in addition to the hearing outcomes. The present results in terms of hearing loss were compared to a review of the literature on the outcomes of malleostapedotomy and/or malleovestibulopexy.

## Results

This report includes 16 patients who have a clinical diagnosis of otosclerosis (*n* = 11), osteogenesis imperfecta (*n* = 1), or with a history of chronic otitis media and or tympanosclerosis (*n* = 4) (Table 2). There were 10 males and 6 females. Indications for a malleostapedotomy and preoperative findings are presented in supplemental table 1. In some cases, there was a combination of erosion of the long process of the incus and an epitympanic fixation of malleus/incus.

**Table 2:** Clinical and audiometric data for the present series of 16 ears with a malleostapedotomy

Nr.	Sex	Age	Year of surgery	Audio follow-up	Etiology	Present number of ear surgeries		Lenght of piston (mm)	Preoperative air–bone gap 0.5–2.0 kHz (dB)	Preoperative air–bone gap 0.5–4.0 kHz (dB)	Postoperative air–bone gap 0.5–2.0 kHz (dB)	Postoperative air–bone–gap 0.5–4.0 kHz (dB)	Postoperative dizziness in first weeks
						Side	Number						
1	♂	57	2005	2 years 3 months	Otosclerosis	Ri	2nd	5.75	28	26	25	26	Almost none
2	♂	65	2005	3 year 9 months	Otosclerosis	Ri	2nd	6.0	47	41	13	10	None
3	♀	46	2006	9 months	Otosclerosis	Le	6th	6.25	60	60	10	14	None
4	♂	58	2006	1 year 2 months	Otosclerosis	Ri	2nd	6.0	17	24	2	4	Almost none
5	♀	45	2006	2 months	Otosclerosis	Ri	3rd	6.25	53	55	12	11	Preop dizziness disappeared
6	♀	48	2006	7 year 11 months	Otosclerosis	Le	2nd	6.25	25	28	3	8	Some in first week
7	♂	42	2007	4 year 8 months	Otosclerosis	Ri	5th	6.25	55	43	10	17	Almost none
8	♀	57	2007	3 year 1 months	Otosclerosis	Ri	1st	5.75	43	43	18	18	Not at all
9	♂	53	2007	8 months	Otosclerosis	Le	4th	6.25	50	53	41–47	41–50	Some in first week
10	♂	56	2007	1 year 7 months	Myringo-sclerosis	Le	3rd	5.75	32	31	5	11	Some in first week
11	♂	51	2008	3 year 2 months	Chronic otitis media	Le	3rd	6.25	52	50	43–47	44–45	None
12	♀	57	2008	9 months	Otosclerosis	Le	3rd	6.25	50	48	10	18	Some only during first 3 months
13	♂	56	2008	2 year 5 months	Chronic otitis media	Ri	6th	6.25	57	51	13	18	None
14	♂	58	2009	9 months	Otosclerosis	Ri	1st	6.25	22	24	10	18	None
15	♀	48	2009	3 year 2 months	Osteog. imp	Ri	3rd	6.5	47	43	7	9	Almost none
16	♂	25	2009	5 years 2 months	Chronic otitis media	Ri	4th	6.5	42	43	5	8	None

In all but one patient general anesthesia has been applied. The surgery in case 4 (Table 2) was combined with a myringoplasty of the postero-superior part of the tympanic membrane by applying autogenous cartilage as an underlay to resolve partial atelectasis involving an eroded incus. The postoperative air–bone gap was 2 dB (0.5–2.0 kHz). Autogenous cartilage was also used as an underlay graft in cases 7, 8 and 13 with a history of chronic otitis media. In all 3 patients, the posterior half of the tympanic membrane was reinforced with a thin slice of autogenous cartilage glued to the medial side of the tympanic membrane to prevent atelectasis.

In this series of 16 ears of a malleostapedotomy with the Kurz–Häusler self-fixing articulated titanium prosthesis, revision surgery was performed in three patients (cases 3, 7 and 15). In all three, the revisions were successful after applying a somewhat longer piston.

The length of all the pistons applied as mentioned on its package is described for each surgery in Table 2. It varied between 5.75 and 6.5 mm. The diameter applied was usually 0.4 mm. In cases of previous total platinectomies the opportunity of a 0.6 mm diameter was used. This applied length of 5.75–6.5 mm, usually 6.25 mm, is longer as the usually applied length of 5.25–5.5 mm as mentioned by Häusler and Steinhart [7]. Even when applying these longer pistons, our postoperative outcomes related to the period of postoperative dizziness (Table 2) were quite good.

Many ears in the present series have had previous ear surgery. This varied between zero and five previous surgeries (Table 2). The audiometric follow-up varied between 8 weeks and 7 years and 7 months. The mean follow-up time was 3 years (Table 2). The pre- and postoperative air–bone gaps for 0.5, 1.0, 2.0 and for 0.5, 1.0, 2.0, 4.0 kHz are separately presented for each case in Table 2. A postoperative air–bone gap equal or less than 10 dB for 0.5, 1.0 and 2.0 kHz was found in 9/16 (56%) cases. An air–bone gap equal or less than 20 dB was found in 13/16 (81%). A comparison with the results as presented in the literature is shown in Table 1. The mean postoperative air–bone gap for this series is 14.3 dB for 0.5–2.0 kHz and 17.3 dB for 0.5–4.0 kHz. Seven other reports on malleostapedotomy results presented also their mean postoperative air–bone gap (Table 3).

**Table 3:** Mean postoperative air–bone gap in dB

Author (reference)	Year	Number of ears	Frequencies			Specification
			0.5–2.0 kHz (dB)	0.5–3.0 kHz (dB)	0.5–4.0 kHz (dB)	
Schuknecht and Bartley [3]	1986	203		14		
Sarac et al. [17]	2006	36		14.3		
Dalchow et al. [18]	2007	6			13	
Kisilevski et al. [20]	2009	24 (20 + 4)		18.6 and 20.7		
Chang et al. [25]	2012	14 = 7 + 7		19.8 and 11.9		
Rambousek et al. [22]	2012	60 = 28 + 32		9.4 and 11.3		
Chang et al. [26]	2014	20	23.5			Handle-malleostapedotomy
		15	22.3			Neck malleostapedotomy
Present series	2014	16	14.3		17.3	

In Table 4, the postoperative air–bone gap levels are presented stratified according to the numbers of previous operations for recent series of *n* = 36 ears from Boston [17] and for the present series. In the present series, even after 3, 4 or 5 previous ear surgeries, equally good outcomes were found with the articulated self-fixing titanium piston.

**Table 4:** Postoperative air–bone gap levels stratified according to numbers of previous operations (Sarac et al. [17], present series)

Number of previous surgeries	None		One		Two		Three		Four		Five	
Centers	Boston (17)	Nijmegen	Boston (17)	Nijmegen	Boston (17)	Nijmegen	Boston (17)	Nijmegen	Boston (17)	Nijmegen	Boston (17)	Nijmegen
Number of ears involved	0/36 (0%)	2/16 (13%)	16/36 (44%)	4/16 (25%)	17/36 (47%)	6/16 (38%)	3/36 (8%)	1/16 (6%)	0%	1/16 (6%)	0%	2/16 (13%)
Air–bone gap ≤ 10 dB	0	1 (6%)	12 (33%)	2 (13%)	3 (8%)	4 (25%)	1 (3%)			1 (6%)		1 (6%)
11–20 dB		1 (6%)	4 (11%)	1 (6%)	7 (19%)	1 (6%)	1 (3%)					1 (6%)
21–30 dB			0	1 (6%)	5 (14%)		0					
> 30 dB			0		2 (6%)	1 (6%)	1 (3%)	1 (6%)				

The immediate postoperative scores for dizziness in the present series are presented in Table 2. These are in the range of the present outcomes for incudo-stapedotomy procedure.

In case 5, there were preoperative complaints of dizziness possibly related to a previously placed piston with a relatively deep position in the vestibulum as seen on CT scan. After revision surgery, the dizziness disappeared fully within days. Besides this, the air–bone gap improved from 53 to 12 dB (0.5, 1.0, 2.0 kHz).

However, the not-applying fat or fibrous tissue on the stapes footplate around the piston as previously propagated by Häusler [9] and Fisch [10] for revision stapes surgeries and malleostapedotomies might even have contributed to the few cases of revision surgeries in this series or showing an incomplete or even lack of success in the case nrs. 1, 9, and 11 in this series.

In cases 7 and 13, after one previous malleostapedectomy, which initially had a good outcome for the hearing, a disconnection of the piston at the level of the footplate had occurred after blowing the nose. The postoperative air–bone gap after revision surgery was 10 dB (0.5–2.0 kHz) for case 7, 18 dB (0.5–2 kHz) for case 8 and 13 dB (0.5–2 kHz) for case 13.

There has been no case of any serious deterioration of the sensorineural component postoperatively (Table 5). For the calculation of the air–bone gap, the postoperative sensorineural hearing levels could, therefore, been used.

**Table 5:** Postoperative changes for 0.5–2.0 kHz and for 0.5–4.0 kHz in bone conduction threshold in dB after malleostapedotomies

Nr.	Preoperative 0.5–2.0 kHz (dB)	Preoperative 0.5–4.0 kHz (dB)	Postoperative 0.5–2.0 kHz (dB)	Postoperative 0.5–4.0 kHz (dB)	Change 0.5–2.0 kHz	Change 0.5–4.0 kHz
1	15	13.75	15	12.50	0	1.25
2	31.6	42.5	35	46.25	− 3.4	− 3.75
3	35	38.75	25	30	10	8.75
4	38.5	41.25	25	28.75	13.5	13.5
5	38.5	38.75	23.3	25	15.2	13.75
6	51.6	52.5	43.4	42.5	8.3	10
7	25	30	20	27.5	5	2.5
8	41.6	36.25	28.3	25	13.3	11.25
9	15	12.5	13.3	17.5	1.7	− 5.5
10	43.3	47.5	31.6	41.25	11.7	6.25
11	46.6	48.75	50	51.25	− 3.33	− 2.25
12	13.3	18.75	10	16.25	3.3	2.5
13	31.6	35	25	27.5	6.6	7.5
14	36.6	45	36.6	45	0	0
15	65	66.25	53.3	56.25	11.7	10
16	10	8.75	8.4	6.25	1.6	2.5

Case 9 and case 11 are the 2 ears with postoperative air–bone gaps larger than 40 dB. Both ears might have lost a few decibels each, but this is considered to be within the variance of such audiometric measurements.

The improvement of the postoperative sensorineural thresholds compared to the preoperative sensorineural thresholds (i.e., ‘overclosure’) in the remaining 14 out of 16 ears varied between 0 and 15.0 dB (0.5, 1.0, 2.0 kHz) and between 0 and 14.0 dB (0.5, 1.0, 2.0, 4.0 kHz) (Table 5), which is indicative for overclosure. The mean improvement was 7 dB HL (0.5–2.0 kHz) and 6 dB HL (0.5–4.0 kHz). Häusler and Oestreicher reported a mean overclosure of 4.3 dB (0.5–3.0 or 0.5–4.0 kHz) in 34 ears. The size of the overclosure varied from 0 to 12.5 dB.

## Discussion

The malleovestibulopexy procedures have been refined over time which has also led to a more accurate terminology, namely malleostapedotomy. The early procedures like the fat-wire piston technique with a total stapedectomy (Sheehy Ref. [1]) and the all-Teflon grip piston with an opening superiorly (Shea Ref. [2]) involved risks for the inner ear function, by rupture and eventually later adhesions of the soft tissue structures in the vestibule. This could also occur by a too deep insertion of the tip of the all-Teflon piston into the vestibule before the piston could be secured to the malleus (Shea Ref. [2]). The Schuknecht piston for a malleostapedectomy procedure required manipulation around the malleus having the tip of the piston already positioned in the vestibule (Refs. [3, 9]). Closure of the piston around the malleus was achieved by bending an extra loop of the piston around the medial aspect of the malleus. For all these procedures considerable percentages of inner ear damage have been reported [1, 3, 9, 11].

In the 80s, the advantages of the incudostapedotomy compared to the incudostapedectomy became well understood and, therefore, as a consequence the naming malleovestibulopexy procedure changed into malleostapedotomy [21]. As part of that procedure an endaural widening incision needed to be combined with supero-anterior widening of the bony ear canal (canalplasty) to improve the feasibility to close the titanium loop around the malleus from laterally.

Once the new self-fixing articulated titanium piston became available, fixation of the piston around the malleus has been simplified [7]. A transcanal approach, using a flexible speculum holder usually provides sufficient access to the middle ear to perform the surgical procedure adequately as has been reported by Häusler and Steinhart [7].

Table 1 shows that over time for the malleovestibulopexy and later on the malleostapedotomy an air–bone gap closure within or equal to 10 dB is possible for 50% of the cases and within or equal to 20 dB for about 80–90% of the cases. Over time, the number of ears with a malleostapedotomy reported in the literature has decreased significantly. The only exception in this is the series published by Fisch et al. [15] and Rambousek et al. [22] both applying the Fisch malleostapedotomy technique combined with a bony canalplasty. Both Swiss groups of authors have reported on larger series possibly because both prefer the malleostapedotomy above the incudostapedotomy procedure in case they have any doubt about the optimal mobility of the malleus incus complex. Therefore, the malleostapedectomy procedure is frequently a primary surgical intervention. Rambousek et al. [22] presented even better hearing outcomes for the group with primary surgery compared to the group with revision surgery.

The results of series presented here fits into the range of results reported in the literature. Over time, the risks for inner ear functions as hearing and balance have decreased so much that these fit into the range known for the incudostapedotomy.

Table 2 for the series reported here shows that the postoperative dizziness noted was none or only very limited for a short while. Even applying relatively longer pistons our postoperative outcomes in terms of postoperative dizziness (Table 2) were quite good.

No remarkable deterioration of the sensorineural hearing level was noted in the series presented here (Table 5). Häusler and Oestreicher [4] also published in detail outcomes for their malleostapedectomy series using the same prothesis. The preoperative thresholds improved in all their successfully operated ears (Table 3).Over time, the risks for inner ear function seem to be decreased considerably and have become comparable to incudostapedotomy.

Our present series shows good results compatible with the standard outcomes as reported earlier in the literature. Explicit are the good outcomes for the vestibular system postoperatively (Table 2). The great advantage of applying the new articulated self-fixing titanium malleostapedotomy piston is that even with a limited transcanal approach, fixation of the piston by its self-fixing mechanism can be realized readily without introduction of the tip of the piston first. As a result of the design with the articulation, the final introduction of the piston may follow as a well-controlled step. Another advantage is that the medial end of the piston will finally have a 90° position to the stapedotomy opening. Especially in ears where the anatomical position (axis of the malleus) related to the oval window niche is quite anteriorly, this articulation helps to bridge that distance and facilitates a position of the stapedotomy opening in the most preferred place at the footplate just a bit inferior or posterior of the center.

This adjustable length of the piston facilitates the application of a somewhat longer piston as sometimes is needed during malleostapedotomy compared to the incudostapedotomy procedure. Having a 90° direct access by the articulation of the piston to the stapedotomy opening in the middle of the footplate and being able to clip the piston first to the malleus before the medial part of the piston is brought into the vestibule will have even especially contributed to that good outcome.

Results like presented in this series might even improve in the next future by applying some additional fat or fibrous tissue with some fibrin glue around the piston on the footplate to help to decrease the risk of a dislocation of the piston at the stapedotomy level. Another surgical possibility is the application of bone cement to the eroded long process of the incus to restore its integrity and make it suitable for a regular stapes piston as was described by Van Rompaey et al.

In conclusion, the outcomes of this series with the new self-fixing articulated titanium piston for the malleostapedotomy procedure are comparable with the surgical outcomes known in the literature for this procedure (Table 1). The simplification of the malleostapedotomy procedure facilitated by the new design of this piston provides a strong argument to expect that this latest step in the development of malleostapedotomy procedures will get a worldwide acceptance.

## Electronic supplementary material

Below is the link to the electronic supplementary material.

Supplementary material 1 (DOCX 19 KB)
