# Supplementary material for: Malleostapedotomy with the self-fixing and articulated titanium piston
Source: Eur Arch Otorhinolaryngol. 2018 May 19;275(7):1715–22. doi: 10.1007/s00405-018-4999-z (PMC5992232; doi:10.1007/s00405-018-4999-z)
Supplement: Supplementary file 1 — Supplementary material 1 (DOCX 19 KB) [file 405_2018_4999_MOESM1_ESM.docx]

**Supplemental table 1. Indications for malleostapedotomy in 16 ears.**

| After previous stapedectomy/stapedotomy   - Ankylosis of incus and/or malleus - Erosion/missing long process of the incus - Luxation of the incus | 6x  10x  1x |
| --- | --- |
| After other otological procedures   - After a previous incus interposition having also a stapes fixation - Revision of a previous malleostapedectomy | 1x  4x |
